# The Effects of 5-Hydroxytryptophan in Combination with Different Fatty Acids on Gastrointestinal Functions: A Pilot Experiment

**DOI:** 10.1155/2014/424503

**Published:** 2014-08-19

**Authors:** Helene Sauer, Isabelle Mack, Silke Kohler, Stefanie Siegle, Nicole Rieber, Stephan Zipfel, Bärbel Otto, Yvonne Ritze, Stephan C. Bischoff, Paul Enck

**Affiliations:** ^1^Department of Psychosomatic Medicine and Psychotherapy, University of Tuebingen, Medical Hospital, Osianderstraße 5, 72076 Tuebingen, Germany; ^2^Department of Internal Medicine IV, University Hospital Munich, Central Campus, Ziemssenstraße 1, 80336 Munich, Germany; ^3^Department of Nutritional Medicine, University of Hohenheim, Fruwirthstraße 12, 70599 Stuttgart, Germany

## Abstract

*Background.* Fat affects gastric emptying (GE). 5-Hydroxythryptophan (5-HTP) is involved in central and peripheral satiety mechanisms. Influence of 5-HTP in addition to saturated or monounsaturated fatty acids (FA) on GE and hormone release was investigated. *Subjects/Methods.* 24 healthy individuals (12f : 12m, 22–29 years, BMI 19–25.7 kg/m²) were tested on 4 days with either 5-HTP + short-chain saturated FA (butter), placebo + butter, 5-HTP + monounsaturated FA (olive oil), or placebo + olive oil in double-blinded randomized order. Two hours after FA/5-HTP or placebo intake, a ^13^C octanoid acid test was conducted. Cortisol, serotonin, cholecystokinin (CCK), and ghrelin were measured, as were mood and GE. *Results.* GE was delayed with butter and was normal with olive (*P* < 0.05) but not affected by 5-HTP. 5-HTP supplementation did not affect serotonin levels. Food intake increased plasma CCK (*F* = 6.136; *P* < 0.05) irrespective of the FA. Ghrelin levels significantly decreased with oil/5-HTP (*F* = 9.166; *P* < 0.001). The diurnal cortisol profile was unaffected by FA or 5-HTP, as were ratings of mood, hunger, and stool urgency. *Conclusion.* Diverse FAs have different effects on GE and secretion of orexigenic and anorexigenic hormones. Supplementation of 5-HTP had no effect on plasma serotonin and central functions. Further studies are needed to explain the complex interplay.

## 1. Introduction

The worldwide prevalence of overweight and obesity is increasing epidemically [1]. The reasons are mainly seen in the growing trend of physical inactivity, coupled with a high caloric intake of easily available and highly palatable energy-rich foods, being generally sweet and/or fatty [2]. The altered eating behaviour of overweight people is frequently associated with delayed gastric emptying and other motility changes of the gut [3]. In addition, food components such as fatty acids (FA), proteins, and carbohydrates influence gastrointestinal conditions and hormone release differently. In obese people, a protein-rich diet resulted in delayed gastric emptying, whereas a high carbohydrate meal accelerated gastric emptying [4]. In general, FA chain length and the ratio between saturated and unsaturated FA have a regulatory effect on gastric emptying, on appetite, and on hormone release [5], for example, the anorexigenic-acting cholecystokinin (CCK) [5] and the orexigenic hormone ghrelin [6].

Another factor discussed in connection with gastric emptying, eating behaviour, and satiety is the amino acid tryptophan, the precursor of serotonin [7, 8]. In the serotonergic pathway, tryptophan is converted to 5-hydroxytryptophan (5-HTP) by the rate limiting enzyme tryptophan hydroxylase. A distinction is made between two isoforms, tryptophan hydroxylase-1, which is formed in several tissues of the body, and tryptophan hydroxylase-2, which is only found in the brain. The enzyme aromatic L-amino acid decarboxylase synthesizes serotonin (5-HT). Máté et al. [9] recently analyzed gene expression during the postnatal development of the myenteric plexus in transgenic mice and found that some neurons simultaneously expressed both CCK and 5-HTP. The authors suggest a possible regulatory role of these special neurons in feeding due to the coexistence of CCK and 5-HTP.

Two established methods exist to investigate the role of tryptophan originating from food on serotonin and its role in regulating central and peripheral functions. Acute tryptophan depletion (ATD) is a temporary reduction of tryptophan and 5-hydroxyindoleacetic acid in plasma and in serotonin availability in the central nervous system [10]. This is achieved by the administration of an amino acid drink that does not contain tryptophan; consequently the neuronal production of serotonin is reduced for hours. This was found to delay gastric emptying as compared to the balanced drink in healthy volunteers [7]. ATD induces typical intestinal symptoms in patients with irritable bowel syndrome (IBS) [11] and also central symptoms in depressed patients [12] and migraine sufferers [13].

The second technique employs the food supplementation of tryptophan or 5-HTP (ATS) in different amounts (500 mg to 5 g), resulting in increased plasma tryptophan levels and availability of serotonin in the central nervous system. In 1989, a study with obese women found that 8 mg/kg 5-HTP daily resulted in a threefold weight loss as compared to a placebo control group [8]. However, the reported effects are less consistent across studies. A delay in gastric emptying among healthy humans following the administration of L-tryptophan was also noted for ATS [14].

Both ATD and ATS exhibit central as well as peripheral (intestinal) effects, raising the issue of whether and how nutritional tryptophan is distributed to the brain and the enteric nervous system in humans. In 1976, Shindo and Miyakoshi [15] analyzed the distribution of orally administered ^14^C-labeled 5-HTP in rats by autoradiographic studies. After a fast intestinal resorption, they found L-5-HTP in the mucosa of the stomach and the gut, in the kidneys, in the pancreas, in the liver, in the spleen, in the skin, and in the central nervous system. Another study by Geeraerts et al. [16] found a significant reduction in serotonin concentrations in the enterochromaffin cells of the human intestine following ATD in comparison to placebo; however, they did not measure central serotonin concentrations.

The aim of this pilot study was to clarify which of the two systems, the central or the enteric nervous system, expresses the higher priority and thus stronger responses under ATS conditions. Due to the faster resorption of 5-HTP in the intestines [15] and the fact that 5-HTP can pass the blood brain barrier without the presence of a transport molecule [17], we administered 5-HTP instead of tryptophan. Tryptophan has to share its transport molecule with other competitive amino acids leading to an inhibited uptake. This was combined with the question whether the fatty acid chain length and the ratio between saturated and unsaturated FAs differentially affect gastric emptying, the circulating levels of anorexigenic and orexigenic hormones, stress hormone release, and whether serotonin is involved in this regulation.

## 2. Methods

### 2.1. Participants and Study Design

Twenty-four healthy participants (12 females, 24.6 ± 1.9 (22–29) years, body mass index (BMI) 22.2 ± 1.6 kg/m^2^) were recruited. All participants gave written informed consent before participating in the study. This randomized, double-blinded placebo-controlled, cross-over study was approved by the Ethical Board of the University of Tuebingen Medical Faculty. No legal registration of the study was required because all used supplements originated from nutritional sources and are not regarded as drugs according to German and European standards.

### 2.2. Protocol

Each participant was investigated on four separate days, with at least three days between two consecutive tests. The sequence of all 4 experimental groups (5-HTP + butter; 5-HTP + olive oil; placebo + butter; placebo + olive oil) was randomized and completely balanced across subjects and groups.

On all four occasions, participants were requested to arrive at the laboratory at 07.45 h, after a 12-hour fast. After taking the first blood sample, participants received two gelatine capsules filled with either 500 mg 5-hydroxytryptophan (5-HTP) or sugar in the placebo group, respectively. The lowest 5-HTP concentration that was normally used in other studies (range from 500 mg to 5 g) was administered in this pilot study, with the idea for a further study testing different concentrations. The participants were asked to stay in the room and refrain from smoking and eating, and physical activity was prohibited.

Two hours later (10.00 a.m.), they received the regular test meal for the ^13^C octanoid acid breath test. It was administered two hours later than the 5-HTP/placebo capsules, because the interesting time point of gastric emptying is 2 h postprandium whereas acute tryptophan depletion occurs 4 h after administration. The test meal consisted of two slices of toast (50 g), one fried egg, 100 *μ*L ^13^C octanoid acid, and 80 mL of orange juice. The juice was enriched with either 40 g of tepid olive oil (long-chain, single-saturated FA) or melted butter (short-chain saturated FA). Furthermore, the FA group was masked in this way. The meals therefore contained about 12.5 g protein and about 32.2 g carbohydrates. Due to the different physical and caloric density of olive oil and butter, the meal with butter included 41.1 g of fat (whole meal: 550 kcal) whereas the meal with olive oil contained 47.6 g fat (whole meal: 609 kcal).

For the next 4 hours, breath samples were taken every 15 minutes for gastric emptying assessment. Saliva samples were taken every hour starting at 9.45 h to assess cortisol release. Blood samples were drawn at 8.00 h (t1), 10.30 h (t2), 12.00 h (t3), and 13.30 h (t4) for measurement of plasma levels of CCK, ghrelin, and serotonin.

Mood was rated on the Profile of Moods States (POMS; adapted German version: ASTS) [18]. The POMS is a 19-item validated test that assesses subjective well-being and has been shown to be sensitive to short-term acute mood changes on five dimensions (sadness, hopelessness, tiredness, positive mood, and anger) and on global negative and positive mood scales [19]. Ratings were taken at 08.15 h, 10.15 h, 11.00 h, 12.00 h, 13.00 h, 14.00 h, and 18.00 h.

After leaving the laboratory, participants were instructed to keep a diary for 24 hours about complications (e.g., nausea, diarrhoea, and changes in taste sensation) and changes in hunger and stool consistency and urgency.

### 2.3. Gastric Emptying

Gastric emptying was measured using the ^13^C octanoid acid breath test technique. It provides similar results compared to the gold standard measurement method for gastric emptying scintigraphy [20]. After passing through the stomach, the stable carbon isotope of the ^13^C octanoid acid is rapidly absorbed in the duodenum. It is then oxidized to ^13^CO_2_ in the liver. The ^13^CO_2_ is immediately exhaled via lungs and can be analyzed in exhaled air collected in special bags. Nondispersive infrared spectroscopy (FANci2, Fischer Analysen Instrumente GmbH, Leipzig, Germany) was used for measuring the ratio of ^13^C-to-^12^C in the CO_2_ in the breath. The half-life (HLF) of gastric emptying was calculated using an approved algorithm [20]. Reference values for the normal rage of HLF were taken from literature [20] and from manufacturer data (according to Wagner Alalysen Thechnik GmbH, 13C breath tests in action).

### 2.4. Blood Samples and Immunoassays

The EDTA blood sample was immediately centrifuged after collection. Plasma supernatant was then transferred into 1.5 mL tubes and frozen at −80°C for later analysis. The concentrations of serotonin (5-hydroxytryptamine, 5-HT), CCK, and ghrelin were assessed in the plasma supernatants by immunoassays. Serotonin was determined by commercial enzyme immunoassay (ELISA) kits according to the manufacturer's instructions (IBL, Hamburg, Germany). CCK was analyzed by radioimmunoassay (RIA) established at the gastroenterology laboratory of the University Hospital, Munich, Germany [21]. It detects all biological active, sulphated molecular forms of CCK with <1% cross-reactivity to sulphated gastrin. The respective coefficients of intra-assay variation were 5.6% and 11.1% and those of interassay variation 12.3% and 13.1%. The same laboratory measured ghrelin using a commercial RIA (Phoenix Pharmaceuticals, Belmont, CA) according to the manufacturer's instructions, using ^125^I-labeled bioactive ghrelin as tracer molecule and polyclonal antibody raised in rabbits against full-length octanoylated human ghrelin (intra-assay coefficient of variation 4%). No cross-reactivity with human secretin, human vasoactive intestinal peptide, human galanin, human GHRH, NYP, or other relevant molecules has been reported [22].

### 2.5. Saliva Samples and Cortisol Analysis

Saliva samples were collected in special tubes (Salivette, Sarstedt AG & Co, Nümbrecht, Germany) to assess salivary cortisol concentrations, which exhibit a good correlation with the unbound cortisol concentration in serum and thus with the biologically active form [23]. A cotton roll was chewed for about one minute and then stored in the Salivette tube. After centrifugation, samples were frozen and later analyzed for the cortisol concentration using a fluorescence immunoassay [24] at the Department of Biological Psychology, Technical University Dresden, Germany.

## 3. Statistics

Kolmogorov-Smirnov tests were used to test for normal distribution. Data were compared between conditions (5-HTP, placebo), fatty acid condition (olive oil, butter) and time points (t1, t2, t3, and t4 for blood parameters) by the 2 × 2 × 4 repeated measure ANOVA, while individual measures were analyzed by 2 × 2 ANOVA. Wilcoxon-single-ranks test was used for the analysis of HLF data between both conditions. Probability values of *P* < 0.05 were considered statistically significant. All analyses were performed by SPSS version 13. All data are presented as means ± SE.

## 4. Results

### 4.1. Gastric Emptying

**Figure 1:**
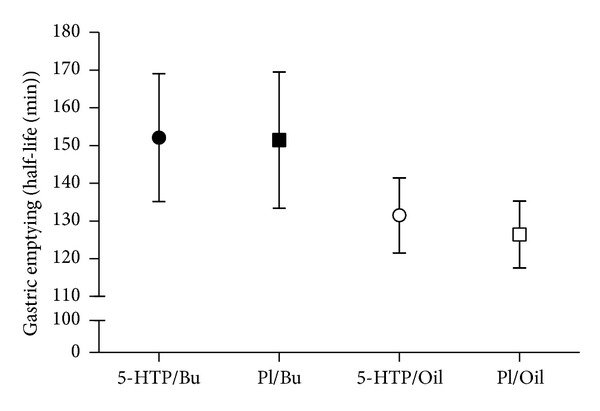
Half-life of gastric emptying. The influence of 5-hydroxytryptophan (5-HTP) and fatty acid ingestion on the half-life (HLF) of gastric emptying. Participants received a capsule with either 5-HTP or placebo (Pl). Additionally they ingested a standard meal supplemented with 100 *μ*L ^13^C octanoid acid and either butter (Bu) or olive oil (Oil). A breath test sample was taken every 15 min over the next 4 hours. The data is presented as means ± SE of the HLF of gastric emptying. Statistics: HLF; no statistical significance within the 4 conditions. There was a significant difference between the summarized butter and olive data, *P* < 0.05.

When values of the oil group and the butter group were summarized regardless of the 5-HTP group, the mean HLF value in the olive oil group was in the normal rage of literature reference values, with 127.3 ± 43 min. The HLF mean value in the butter group differed significantly from the olive oil group HLF value (*P* < 0.05) and was, with 153.9 ± 72 min and compared to literature values, delayed. As the normal range depends on the administered food composition, the measured values of the placebo conditions in our study have to be regarded as reference values. Mean HLF was 152 ± 68 min for 5-HTP + butter, 151 ± 77 min for placebo + butter, 132 ± 45 min for 5-HTP + oil, and 126 ± 42 min for placebo + oil (Figure 1). There were no statistically significant differences between the groups and also no influence of 5-HTP supplementation.

### 4.2. Serotonin, CCK, and Ghrelin Plasma Levels

**Figure 2:**
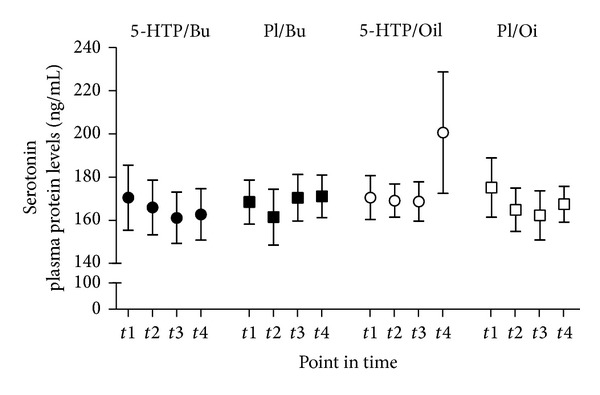
Serotonin plasma levels. The influence of 5-hydroxytryptophan (5-HTP) and fatty acid ingestion on serotonin plasma concentrations. Participants received a capsule with either 5-HTP or placebo (Pl). They also ingested a standard breakfast supplemented with 100 *μ*L ^13^C octanoid acid and either butter (Bu) or olive oil (Oil). Plasma samples were taken during fasting (t1) and after 5-HTP or placebo intake after 2.5 hrs (t2), 4 hrs (t3), and 5.5 hrs (t4). The actual plasma concentrations of serotonin protein are presented as means ± SE. Statistics: serotonin; no statistical significance.

Serotonin concentrations remained unchanged after 5-HTP (or placebo) supplementation for each of the fatty acid groups (Figure 2).

**Figure 3:**
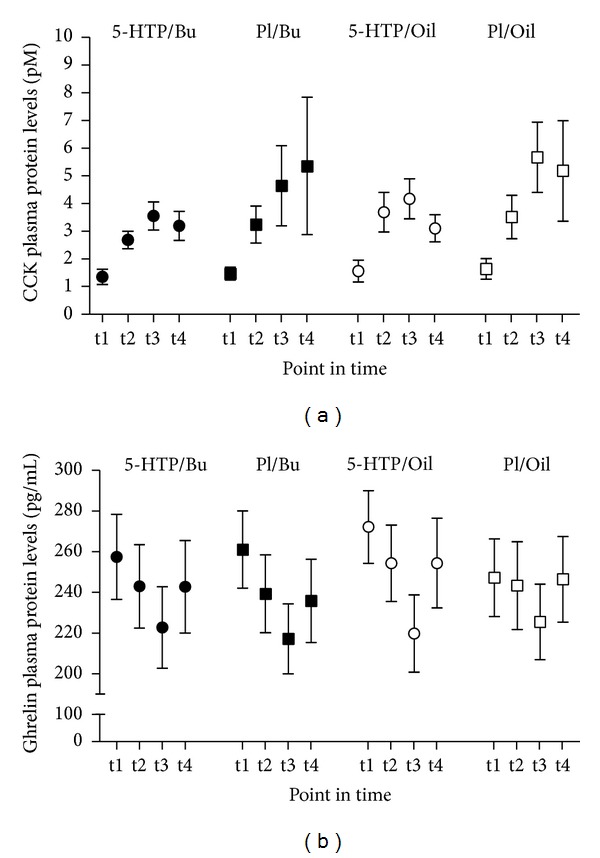
Cholecystokinin and ghrelin. The influence of 5-hydroxytryptophan (5-HTP) and fatty acid ingestion on cholecystokinin (CCK (a)) and ghrelin (b) plasma concentrations. Participants received a capsule with either 5-HTP or placebo (Pl). They also ingested a standard breakfast supplemented with 100 *μ*L ^13^C octanoid acid and either butter (Bu) or olive oil (Oil). Plasma samples were taken during fasting (t1) and after 5-HTP or placebo intake after 2.5 hrs (t2), 4 hrs (t3), and 5.5 hrs (t4). In (a) and (b) the actual plasma concentrations of CCK and ghrelin protein are presented as means ± SE. Statistics: CCK; time:* F* = 6.136; *P* < 0.05. Ghrelin; fatty acid*5-HTP/Pl:* F* = 9.166; *P* < 0.001.

As expected, CCK plasma levels increased after the meal and reached a maximum at 120 min (t3) after meal administration (*F* = 6.136; *P* < 0.05; Figure 3(a)). The elevated CCK values at 120 min (t3) and 210 min (t4) after meal administration did not reach significance due to high standard deviations. In contrast, plasma ghrelin concentrations decreased after the meal, reaching their minimum at 120 min (t3) and rising again at 210 min (t4) postprandially (*F* = 10,038; *P* < 0.001). Adjusting the individual ghrelin data to the respective fasting values at t1 (Figure 3(b)) resulted in a significant interaction of 5-HTP supplementation and fatty acids. The plasma ghrelin levels were significantly reduced in the 5-HTP + olive oil group (*F* = 9.166; *P* < 0.001) but not under 5-HTP + butter.

### 4.3. Cortisol Saliva Levels

**Figure 4:**
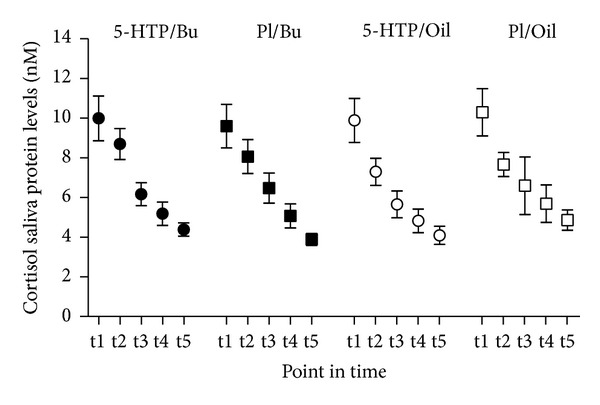
Cortisol. The influence of 5-hydroxytryptophan (5-HTP) and fatty acid ingestion on the circadian profile of saliva cortisol concentrations. Participants received a capsule with either 5-HTP or placebo (Pl). They also ingested a standard breakfast supplemented with 100 *μ*L ^13^C octanoid acid and either butter (Bu) or olive oil (Oil). Saliva samples were taken during fasting (t1 at 8:00) and after 5-HTP or placebo intake after 1:45 hrs (t2), 2:45 hrs (t3), 3:45 hrs (t4), and 4:45 hrs (t5). The actual saliva concentrations of cortisol protein are presented as means ± SE. Statistics: cortisol; time: (*F* = 53.814; *P* < 0.001).

As expected, a significant daytime-related decrease in the cortisol levels was measured (*F* = 53.814; *P* < 0.001) (Figure 4). Neither the supplementation of 5-HTP nor administration of fatty acids had any influence on the cortisol levels.

### 4.4. Satiety, Stool, and Mood Rating

**Figure 5:**
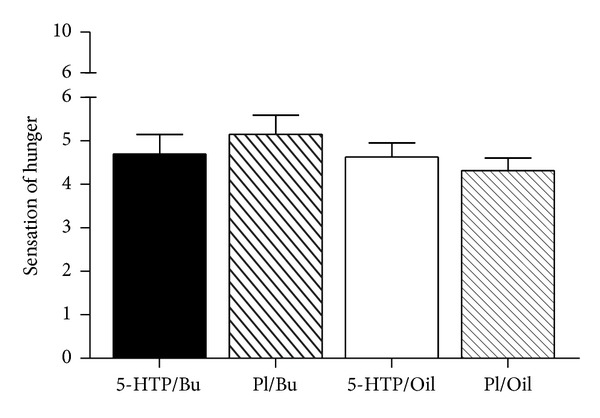
Hunger rating. The influence of 5-hydroxytryptophan (5-HTP) and fatty acid ingestion on the sensation of hunger. Participants received a capsule with either 5-HTP or placebo (Pl). They also ingested a standard breakfast supplemented with 100 *μ*L ^13^C octanoid acid and either butter (Bu) or olive oil (Oil). Over the next 24 hours, the sensation of hunger was recorded using a scale ranging between 0 (no sensation of hunger) and 10 (strong sensation of hunger). The data are presented as means ± SE. Statistics: no statistical significance.

Neither the administration of different fatty acids nor the supplementation of 5-HTP had any influence on the POMS scales for sadness, hopelessness, tiredness, positive mood, and anger (data not shown). It also did not affect hunger (Figure 5) and stool ratings (data not shown) within the first 24 hours after the test.

## 5. Discussion

Our study gained new insight into the influence of different fatty acid conditions (short-chain saturated or monounsaturated) in combination with 5-HTP on gastric emptying and hormone release. We found (1) that the administration of 40 g butter resulted in a delayed gastric emptying; however, the HLF in the olive oil group was in the normal range. Supplementation of 5-HTP showed no effect. (2) Neither the administration of FA nor 5-HTP supplementation influenced serotonin plasma levels. (3) Following the administration of the fatty meals, CCK plasma levels increased over time and caused a decrease in the ghrelin plasma levels. This decrease was significantly different in the 5-HTP + olive oil group. (4) The diurnal profile of cortisol in saliva was unaffected by the administration of FA and 5-HTP supplementation, as were mood, hunger, and stool ratings.

The aim of the present study was to clarify the priority ratio of serotonin in the two different nervous systems, CNS (changes in mood) and ENS (HLF), under different conditions. In our case, no changes in mood/hunger/stool could be observed, but gastric emptying was delayed in the butter group and at the end of the normal range in the olive oil group. This may be best explained by the caloric content and the composition of our test meal. It contained 40 g fat and had a caloric content of 550 to 608 kcal. Hunt and Stubbs showed that the HLF depends on the caloric content (longer after 550 kcal compared to 250 kcal). Additionally, a high fat content leads to a delay in gastric emptying [25] and causes, among other effects, the release of CCK which is responsible for an inhibition of gastric emptying [26].

### 5.1. Gastric Emptying and Serotonin Metabolites

In this study, we did not find any influence of supplemented 5-HTP, the precursor of serotonin, on the HLF. To our knowledge, there are no studies analyzing the effect of the oral administration of 5-HTP in humans in connection with gastric emptying. In dogs, 5-HTP increased gastric motility but had no influence on gastric emptying [27]. In humans, the intravenous administration of 5-HTP resulted in an inhibition of gastric motility, whereas gastric emptying was not investigated [28]. Interestingly, the results after the administration of L-tryptophan are contrary to the findings with 5-HTP. Van Nieuwenhoven et al. found a significantly delayed gastric emptying in eight of ten healthy women during an ATD condition compared to placebo (HLF ATD: 137.2 min; HLF placebo: 98.5 min). They hypothesized that alterations in gastrointestinal motility might be manipulated by nutritional factors in healthy subjects [7].

With olive oil/5-HTP and /placebo, gastric emptying time was near the normal range suggesting that more 5-HTP could be absorbed faster. Nonetheless, the amount of absorbed 5-HTP could not change serotonin plasma levels. However, it is imaginable that there are changes at the level of duodenal serotonin production. About 90 percent of body's serotonin is accumulated in the gut [29], 95 percent of which is stored in the secretory granules of enterochromaffin cells [30]. Keszthelyi et al. conducted a study in which they analyzed the effect of oral administered 5-HTP (100 mg) on intestinal barrier function and mucosal 5-HT metabolism in 15 healthy volunteers. They found that 5-HTP levels in tissue samples of the duodenal mucosa were significantly increased, but 5-HT levels were unaffected [31]. These changed 5-HTP levels may be responsible for the delay in gastric emptying and may regulate the secretion of gastric hormones.

### 5.2. Influence of Serotonin Metabolites and Fatty Acids on Hormone Release [32]

In our present study, we administered butter and olive oil. According to Baltes, olive oil mainly consists of fatty acids with more than 16 carbon atoms, while butter contains fatty acids with less carbon atoms [32]. Assuming that the preabsorptive effects of the fatty acid composition of the different fats result in various diverse changes in hormone secretion, this can be an explanation for the significant reduction in the plasma ghrelin levels in our olive oil group. In our present case, however, this effect only occurs in the 5-HTP plus olive oil group and is nearly completely absent in the placebo/olive oil group. Therefore, in the present study, 5-HTP supplementation also has to play a distinct role regarding the regulation of hormone concentrations. Zhang et al. explored the influence of oral infusion of tryptophan in comparison to 5-HTP and saline on ghrelin expression, food intake, and weight gain among weanling pigs. It was revealed that plasma ghrelin levels were highest after tryptophan administration. After the 5-HTP infusion, food intake was lower than after taking saline; however, tryptophan induced an increase in food intake. It was concluded that tryptophan administration increases ghrelin expression in the gastric fundus and the plasma ghrelin levels [33]. In another animal study, Tena-Sempere and colleagues showed that ghrelin has an inhibiting effect on prolactin release, independent of 5-HTP. They concluded that ghrelin inhibits prolactin secretion without the influence of the nitric oxide, dopamine, and serotonin systems [34].

In our study we not only found—as expected—a decrease in plasma ghrelin after a meal and 5-HTP/placebo administration, but also found an interaction of the 5-HTP supplementation and FAs. In all four groups, ghrelin levels decreased after consuming the meal due to the suppressing effect on ghrelin secretion by the ingestion of carbohydrates and fat [35]. However, a maximum decrease in ghrelin was observed in the 5-HTP + olive oil group two hours after the administration of the test meal. Therefore, we propose that the chain length of fatty acids in a meal differently influences ghrelin secretion.

Similarly, Feltrin et al. analyzed the influence of various fatty acid chain lengths on different gut hormone levels. They revealed that dodecanoic acid (C12) and decanoic acid (C10) both stimulated CCK secretion, but ghrelin plasma levels only significantly decreased in the C12-acid group. They concluded that the effects of fatty acids on ghrelin have to be dependent on their chain lengths and on the different pathways of absorption. The authors suggested that preabsorptive factors might be responsible for the different ghrelin levels after the ingestion of C12 and C10 fatty acids [6]. Furthermore, the release of CCK increased in the presence of fat which may be responsible for the inhibition of gastric emptying [36]. In addition, it has been shown that CCK and serotonin are linked in the control of satiety and that tryptophan treatment leads to a significant decrease in CCK concentrations in the hypothalamus of rats [37].

### 5.3. Limitations of the Study

We need to acknowledge several limitations of this pilot study. Firstly, a more differentiated rating of hunger would have allowed assessment of hunger and satiety in parallel with mood ratings and other measures of central nervous system (CNS) functions in parallel with mood. Kunisato et al. performed a resting-state fMRI study with 21 healthy men in which they showed that changes in some subscores of the POMS correlated with changes in orbitofrontal cortex (OFC) activity of the brain under ATD. Less activity of the OFC was associated with an increase of depressive mood during the depletion situation [38]. To the best of our knowledge, there are no imaging studies which investigate the influence of orally administered 5-HTP supplementation on the brain.

Whilst we did not assess blood 5-HTP levels, routine assessment of tryptophan and its metabolites in tissue samples from the gut appears to be too invasive for a pilot study but may be considered in subsequent investigations.

## 6. Conclusion

In summary, different fatty acids appear to have different effects on gastric emptying and the release of orexigenic (ghrelin) and anorexigenic (CCK) hormones, and 5-HTP supplementation has no effects on core CNS functions, serotonin plasma levels, and cortisol salvia. This can be interpreted in two different ways. Either the observed peripheral effects on the intestine, such as changes in hormone levels, are directly influenced by the increased amount of tryptophan and the various fatty acids, or serotonin production in the enterochromaffin cells is induced by 5-HTP supplementation, despite unchanged plasma serotonin levels, and the increased concentration of serotonin in the cells of the gut affects the secretion of gastric hormones as well as the delay of gastric emptying induced by the fatty acids. The second interpretation seems more likely but would need independent validation in future studies.
